# Supplementary figures and images for: Automatic detection of teacher behavior in classroom videos using AlphaPose and Faster R-CNN algorithms
Source: PeerJ Comput Sci. 2025 May 30;11:e2933. doi: 10.7717/peerj-cs.2933 (PMC12193412; doi:10.7717/peerj-cs.2933)

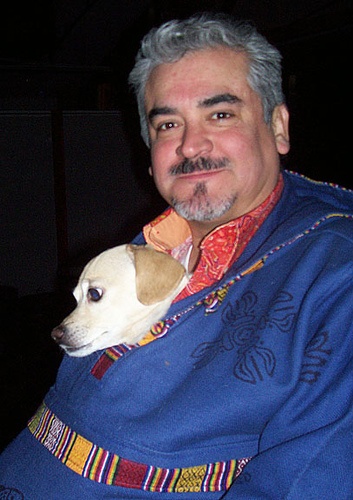

Supplement: Supplemental Information 1 [file peerj-cs-11-2933-s001.zip › code/images/img1.jpg]

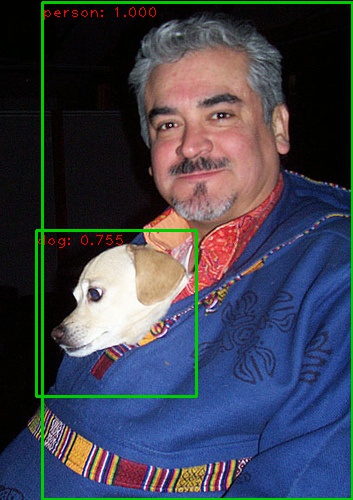

Supplement: Supplemental Information 1 [file peerj-cs-11-2933-s001.zip › code/images/img1_det.jpg]

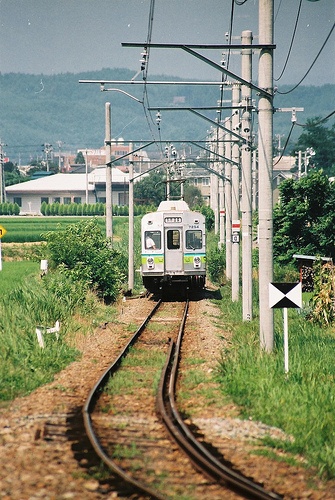

Supplement: Supplemental Information 1 [file peerj-cs-11-2933-s001.zip › code/images/img2.jpg]

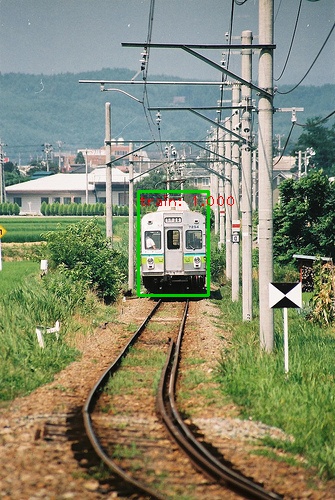

Supplement: Supplemental Information 1 [file peerj-cs-11-2933-s001.zip › code/images/img2_det.jpg]

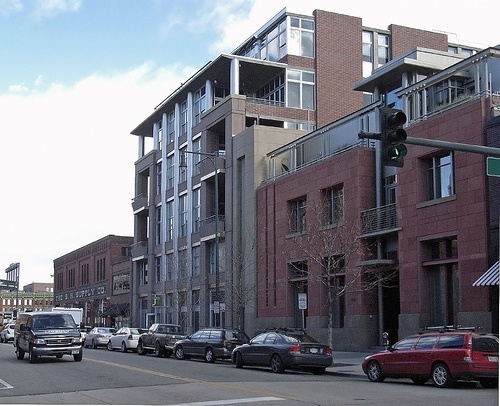

Supplement: Supplemental Information 1 [file peerj-cs-11-2933-s001.zip › code/images/img3.jpg]

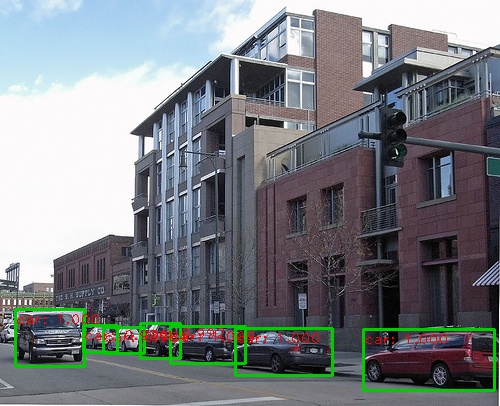

Supplement: Supplemental Information 1 [file peerj-cs-11-2933-s001.zip › code/images/img3_det.jpg]

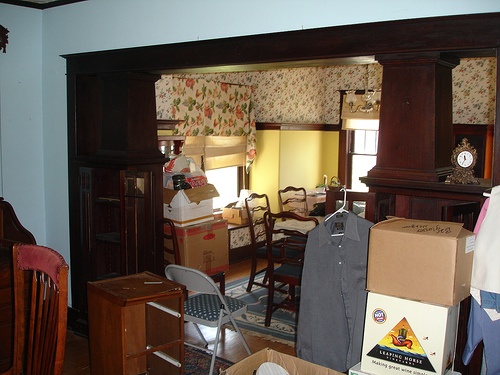

Supplement: Supplemental Information 1 [file peerj-cs-11-2933-s001.zip › code/images/img4.jpg]

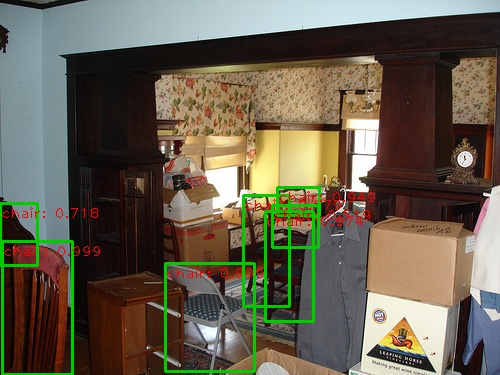

Supplement: Supplemental Information 1 [file peerj-cs-11-2933-s001.zip › code/images/img4_det.jpg]
